# Non-Vitamin K Oral Anticoagulant After Transcatheter Aortic Valve Replacement: A Systematic Review and Meta-Analysis

**DOI:** 10.3389/fphar.2022.755009

**Published:** 2022-02-11

**Authors:** Dongxu Li, Xiaofang Ma, Xu Zhou, Yongjun Qian

**Affiliations:** ^1^ Department of Cardiovascular Surgery, West China Hospital, Sichuan University, Chengdu, China; ^2^ Department of Emergency Medicine, Emergency Medical Laboratory, West China Hospital, Sichuan University, Chengdu, China; ^3^ Evidence-based Medicine Research Center, Jiangxi University of Traditional Chinese Medicine, Nanchang, China; ^4^ National Clinical Research Center for Geriatrics, West China Hospital, Sichuan University, Chengdu, China

**Keywords:** transcatheter aortic valve replacement, non-vitamin K oral anticoagulant, mortality, bleeding, stroke

## Abstract

**Objective:** We aimed to compare non-vitamin K oral anticoagulants (NOACs) with a traditional antithrombotic such as vitamin K antagonist (VKA) and antiplatelet agents in patients after transcatheter aortic valve replacement (TAVR).

**Methods:** We conducted a search in PubMed, EMBASE, and the Cochrane Library until November 2021 for studies involving comparisons of any type of NOACs, including dabigatran, apixaban, rivaroxaban, and edoxaban, with VKA or antiplatelet agents after TAVR. A comparison of NOACs versus VKA was performed in patients with an indication for oral anticoagulation. In addition, we compared NOACs versus antiplatelet in patients without such indication. We calculated the hazard ratios with 95% confidence intervals (CIs) to determine long-term outcomes. The primary outcome was a combined endpoint consisting of all-cause mortality, stroke, major bleeding, or any related clinical adverse events. Secondary outcomes were all-cause mortality, major bleeding, and stroke, respectively.

**Results:** A total of 10 studies including 10,563 patients after TAVR were included in this meta-analysis. There were no significant differences in any of the long-term outcomes between the NOAC and VKA groups. Although there were no significant differences in the combined endpoint, major bleeding, or stroke, a significant difference was observed in the all-cause mortality (HR 1.74, 95% CI 1.25–2.43, *p* = 0.001) between the NOAC and antiplatelet groups.

**Conclusion:** For patients with an indication for oral anticoagulation after TAVR, NOACs seem to be associated with noninferior outcomes compared with VKA therapy. However, for patients without an indication for oral anticoagulation, NOACs appear to be associated with a higher risk of all-cause death as compared with antiplatelet treatment.

**Systematic Review Registration:** https://clinicaltrials.gov/, identifier CRD42020155122.

## Introduction

Since the introduction of transcatheter aortic valve replacement (TAVR) in 2002, it has been widely used in high-risk patients with aortic stenosis (Cribier et al., 2002; Hamm et al., 2016). In addition, the consequent antithrombotic therapy after TAVR has remained an important issue (Sun et al., 2009; Guedeney et al., 2019). Since thrombosis may originate from the valved stent, bioprosthetic valve, or other related diseases (Trepels et al., 2009; Tay et al., 2011; De Marchena et al., 2015; Piayda et al., 2018; Mangieri et al., 2019; Ranasinghe et al., 2019), there are currently two main antithrombotic strategies, namely, antiplatelet therapy and anticoagulation therapy (Sherwood and Vora, 2018; Lugo et al., 2020).

In patients with valvular heart disease, the main indications for anticoagulation include chronic or paroxysmal atrial fibrillation, lung embolism, deep vein thrombosis, poor left ventricular ejection fraction (including left ventricle aneurysms), and extensive arterial vascular disease (Chesebro et al., 1986; Figini et al., 2013; Nijenhuis et al., 2020). Thus, recent guidelines from the American College of Cardiology (ACC) and European Society of Cardiology (ESC) have divided patients who have undergone TAVR into those who have an indication for oral anticoagulation and those who do not have an indication (Falk et al., 2017; Otto et al., 2021).

For patients without an indication, the ACC (Otto et al., 2021) recommends “aspirin 75–100 mg daily is reasonable in the absence of other indication for oral anticoagulants (moderate recommendation); or dual antiplatelet therapy with aspirin 75–100 mg and clopidogrel 75 mg may be reasonable for 3–6 months (weak recommendation); or anticoagulation with a VKA to achieve an INR of 2.5 in patients at low risk of bleeding for at least 3 months (weak recommendation),” whereas the ESC (Falk et al., 2017) recommends “dual antiplatelet for the first 3–6 months followed by lifelong single antiplatelet, or single antiplatelet in the case of high bleeding risk.” For patients with an indication, no specific recommendation is found in the ACC guideline, whereas the ESC recommends lifelong oral anticoagulation therapy. Aside from the consensus on 3-to-6-month dual antiplatelet therapy for TAVR patients who do not need oral anticoagulation, a detailed recommendation of oral anticoagulation for TAVR patients, especially for patients with an indication, remains unclear.

There are currently four non-vitamin K oral anticoagulants (NOACs) approved in clinical therapy, including dabigatran, apixaban, rivaroxaban, and edoxaban (Angiolillo et al., 2018; Levy et al., 2018). Because of the advantages of their shorter half-life, less drug interaction, and no requirement for repeated measurement of the international normalized ratio, NOACs have been used as the first-line drug for patients with nonvalvular atrial fibrillation and deep vein thrombosis (Verheugt and Granger, 2015; Diener et al., 2017; Steffel et al., 2018; Ortel et al., 2020). However, the application of NOACs in patients after TAVR is still controversial (Nijenhuis et al., 2019; Saito et al., 2020). Thus, we aimed to conduct a systematic review and meta-analysis to assess the outcomes of NOACs versus VKA and NOACs versus antiplatelets in patients after TAVR, with the aim of providing some evidence for a clinical treatment strategy.

## Methods

### Registration and Study Protocol

This study was conducted according to the Preferred Reporting Items for Systematic Reviews and Meta-Analyses guidelines (Supplementary File S1) (Moher et al., 2009). The study was registered in the PROSPERO international prospective registry of systematic reviews (CRD42020155122).

### Search Strategy

A literature search prior to November 15, 2021, was conducted in PubMed, EMBASE, and the Cochrane Library databases using predefined medical subject heading terms, Boolean operators, and truncation symbols in combination with direct keywords. The detailed search strategies were as follows: “((oral anticoagulant*) OR (DOAC*) OR (NOAC*) OR (Dabigatran) OR (Apixaban) OR (Rivaroxaban) OR (Edoxaban)) AND ((transcatheter aortic valve) OR (TAVR) OR (TAVI)).” To ensure a complete search, the reference lists of the identified studies were independently reviewed by two authors (D.X.L. and X.F.M.).

### Inclusion and Exclusion Criteria

All included studies were either randomized controlled trials (RCTs) or observational studies that reported the baseline characteristics of patients. The inclusion criteria were as follows: 1) studies assessing at least one kind of NOAC, such as dabigatran, apixaban, rivaroxaban, and edoxaban; 2) studies comparing the effects of NOACs with vitamin K antagonist (VKA) or antiplatelets in patients who had undergone TAVR; and 3) studies reporting at least one of the following variables after agent administration: any kind of endpoint event, death, bleeding, or stroke. Abstracts with complete information were also included. In addition, we excluded animal studies, case reports, and articles for which the full text was not available in English.

### Risk of Bias Assessment

A risk of bias assessment was conducted for all included studies. The Cochrane risk of bias tool was used for RCTs, and the risk of bias in non-randomized studies-of interventions (ROBINS-I) tool was used for non-RCTs (Higgins et al., 2011; Sterne et al., 2016). Both tools were evaluated with eight categories, respectively. Each domain was judged as high, low, or unclear risk of bias with the overall assessment of each study graded as low risk of bias (when more than five domains were low risk of bias), high risk of bias (at least three domains were high risk of bias), or medium risk of bias (otherwise).

### Data Extraction and Outcomes of Interest

Full texts of all included studies were reviewed, and data extraction was performed by two independent authors (D.X.L. and X.F.M.), with disagreement resolved by a consensus among all authors. The characteristics of the studies included publication year, study region, study design, sample size, age, sex, body mass index, any kind of risk score for cardiovascular surgery such as the Society of Thoracic Surgeons (STS) score, the risk score for stroke for anticoagulation such as the CHA_2_DS_2_-VASc score, type of bioprosthetic valve, related medication, and follow-up period. In addition, previously related diseases in the patients included atrial fibrillation, hypertension, diabetes mellitus, renal dysfunction, coronary artery disease, stroke, intracerebral bleeding, and arrhythmia that required a permanent pacemaker. In the STS score system, 0%–4% refers to low risk, 4–8% to moderate risk, and >8% to high risk (Ishizu et al., 2021). In the CHA_2_DS_2_-VASc score, 1–2 points indicate low risk, 3–4 points moderate risk, and >5 points high risk (Jacobs et al., 2015). The main outcome was the combined endpoint event (a composite of all-cause mortality, stroke, major bleeding, or any related clinical adverse events including acute kidney injury, coronary obstruction, major vascular complications, and valve dysfunction requiring reintervention). Additional outcomes were all-cause mortality, major bleeding (including life-threatening and disabling bleeding), and stroke, respectively. All of the above-mentioned outcomes were long-term outcomes with follow-up time and were extracted as time-to-event data.

### Statistical Analysis and Meta-analysis

We compared NOACs versus VKA and NOACs versus antiplatelets according to whether the patient had an indication or not for oral anticoagulation, separately. Subgroup analyses were stratified by the research type. The outcomes of interest were extracted directly from original studies as the hazard ratio (HR) accompanied by the 95% confidence interval (CI) and were pooled by the inverse variance method with a random-effects model (DerSimonian and Kacker, 2007). Statistical heterogeneity was tested using the chi-square test and I^2^ test. If the result of an analysis resulted in *p* < 0.05 or I^2^ > 50%, the studies were considered to be heterogeneous. To explore the source of heterogeneity, if necessary, sensitivity analysis was conducted. When more than 10 studies were included in the meta-analysis, a funnel plot with Egger’s regression test was performed to detect any potential publication bias (Higgins and Green, 2011). All statistical analyses were performed assuming a two-sided test at 5% level of significance, using Review Manager software (version 5.4.1; Cochrane Collaboration, Oxford, United Kingdom).

## Results

### Search Results and Study Characteristics

**FIGURE 1:**
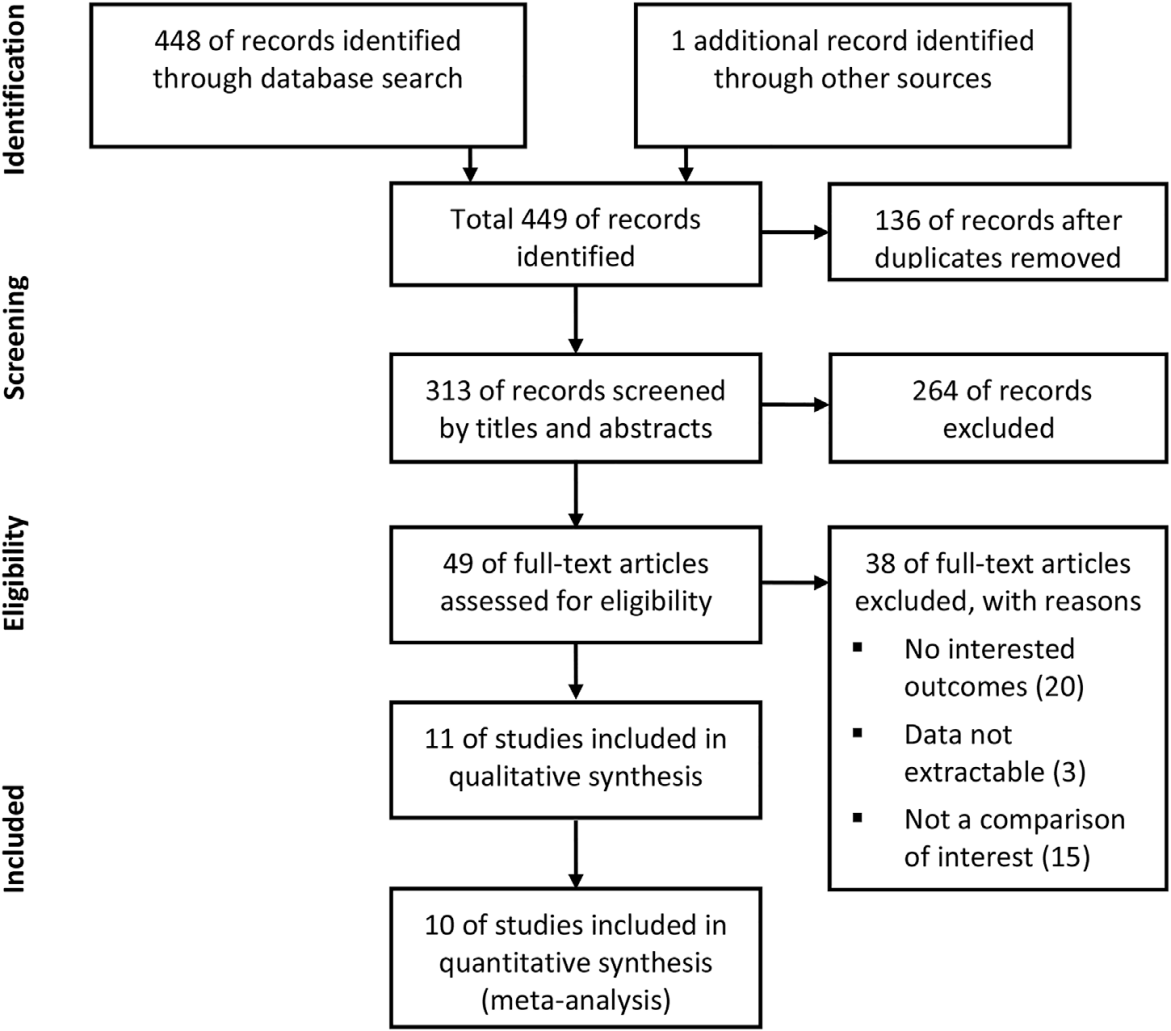
Flow diagram of the literature search and study selection.

According to the inclusion and exclusion criteria, 11 studies were included in the qualitative analysis (Seeger et al., 2017; Geis et al., 2018; Jochheim et al., 2019; Kosmidou et al., 2019; Dangas et al., 2020; Kalogeras et al., 2020; Kawashima et al., 2020; Butt et al., 2021; Collet, 2021; Didier et al., 2021; Van Mieghem et al., 2021). Because one study did not report outcomes of interest as the HRs we needed, 10 studies consisting of 10,563 patients who underwent TAVR were included in the meta-analysis. The detailed steps of the literature search are presented in the flow diagram in Figure 1. Table 1 shows the characteristics of the included studies and patient baseline characteristics. Ten studies included the comparison of NOACs versus VKA in TAVR patients with an indication for oral anticoagulation. Two studies included the comparison of NOACs versus antiplatelet in patients without an indication.

**TABLE 1:** Characteristics of included studies.

Author	Year	Region	Design	Group	Medication	N	Age, year	Sex, M, %	BMI, kg/m^2^	STS score *, %	CHA_2_DS_2_-VASc score †	Endpoint ‡, n	Mortality, n	Bleeding, n	Stroke, n	Follow- up, months
Seeger et al. (2017)	2017	Germany	PC	NOAC	Api+ 4-week SAPT/DAPT	141	82.1 ± 5.3	49.6	27.2 ± 4.2	7.5 ± 5.2	5.0 ± 1.2	22	19	NA	1	12
				VKA	Warf+ 4-week SAPT/DAPT	131	80.5 ± 6.3	51.9	27.4 ± 5.1	7.9 ± 6.3	4.9 ± 1.1	9	6	NA	1	12
Geis et al. (2018)	2018	Germany	RC	NOAC	Dabi/Riva/Api/Edo	154	83.1 ± 5.3	49.4	26.6 ± 5.3	4.1 ± 1.9	4.6 ± 1.2	17	12	3	5	6
				VKA	Warf	172	83.0 ± 4.9	45.3	27.0 ± 5.3	4.4 ± 2.4	4.8 ± 1.3	14	11	3	2	6
Jochheim et al. (2019)	2019	Germany	PC, PSM	NOAC	Riv/Api/Dabi + less than 3-month SAPT/DAPT	326	81.6 ± 6.7	47.9	26.3 ± 5.2	4.5 ± 1.2	NA	63	47	69	10	12
				VKA	Warf + less than 3-month SAPT/DAPT	636	81.1 ± 6.1	47.3	26.6 ± 4.9	4.5 ± 1.2	NA	87	70	146	13	12
Butt et al. (2021)	2019	Denmark	RC, PSM	NOAC	Dabi/Riva/Api+ 6-month SAPT/DAPT	219	83 ± 1.2	53.9	NA	NA	5.0 ± 1.4	NA	15	11	NA	12 ± 1
				VKA	Warf+ 6-month SAPT/DAPT	516	82 ± 1.3	53.7	NA	NA	4.9 ± 1.3	NA	54	28	NA	27.4 ± 1
Kosmidou et al. (2019)	2019	United States	RC, PSM	NOAC	Dabi+ 6-month SAPT/DAPT	155	82.8 ± 6.7	65.6	28.4 ± 6.1	8.2 ± 4.2	5.6 ± 1.3	39	33	8	12	33.6 ± 3.6
				VKA	Warf+ 6-month SAPT/DAPT	778						234	207	43	41	
Kalogeras et al. (2020)	2019	United Kingdom	RC, PSM	NOAC	Dabi/Riva/Api/Edo+ in-hospital SAPT/DAPT	115	81.9 ± 6.3	59.1	27.3 ± 5.8	NA	NA	13	13	NA	NA	15.1 ± 3.8
				VKA	Warf+ in-hospital SAPT/DAPT	102	82.5 ± 5.8	57.8	25.9 ± 5.8	NA	NA	16	16	NA	NA	15.1 ± 3.8
Kawashima et al. (2020)	2020	Japan	PC, PSM	NOAC	Dabi/Riva/Api/Edo + SAPT/DAPT	227	84.4 ± 4.7	30.4	22.6 ± 3.8	7.7 ± 5.1	5.1 ± 1.0	NA	NA	NA	NA	19 ± 2.5
				VKA	Warf + SAPT/DAPT	176	84.3 ± 4.9	36.9	21.7 ± 3.7	9.5 ± 9.5	5.2 ± 1.1	NA	NA	NA	NA	
Dangas et al. (2020)	2020	Switzerland	RCT, ITT	NOAC	Riva+ 3-month aspirin	826	80.4 ± 7.1	51.6	28.1 ± 5.5	4.0 ± 3.2	4.5 ± 1.3	105	64	46	30	14.3 ± 2.3
				Antiplatelet	Aspirin+ 3-month clopidogrel	818	80.8 ± 6.0	49.5	28.2 ± 5.7	4.3 ± 3.5	4.6 ± 1.2	78	38	31	25	15.8 ± 1.7
Didier et al. (2021)	2021	France	RC, PSM	NOAC	Dabi/Riva/Api/Edo+ in-hospital SAPT/DAPT	1,378	83.4 ± 6.1	52.6	27.1 ± 5.5	NA	NA	NA	161	55	29	13.0 ± 2.4
				VKA	Warf+ in-hospital SAPT/DAPT	1,093	83.5 ± 6.4	51.9	26.9 ± 5.1	NA	NA	NA	263	91	37	21 ± 3.4
Van Mieghem et al. (2021)	2021	Multiple countries	RCT, ITT	NOAC	Edo+ 3-month SAPT/DAPT	713	82.1 ± 5.4	51.3	27.5 ± 5.7	4.8 ± 3.5	4.5 ± 1.4	170	85	98	29	18.5
				VKA	Warf+ 3-month SAPT/DAPT	713	82.1 ± 5.5	53.6	27.9 ± 5.4	5.0 ± 4.1	4.5 ± 1.3	157	93	68	35	17.7
Collet (2021)	2021 (Presentation)	France	RCT, ITT	NOAC	Api	749	81.6 ± 6.1	45.9	27.5 ± 5.5	5.1 ± 5.0	4.4 ± 1.4	64	54	64	28	12
				VKA/Antiplatelet	Warf/SAPT + DAPT	228/523	82.3 ± 6.4	47.9	27.3 ± 5.2	5.1 ± 5.4	4.3 ± 1.4	64	41	64	21	12

M: male; BMI: body mass index; PC: prospective cohort: RC: retrospective cohort; PSM: propensity score matching; RCT: randomized controlled trial; ITT: intention to treat; NOAC: non-vitamin K oral anticoagulant; VKA: vitamin K antagonist; Api: apixaban; Warf: warfarin; Dabi: dabigatran; Riva: rivaroxaban; Edo: edoxaban; SAPT: single-antiplatelet therapy; DAPT: dual-antiplatelet therapy; NA: not applicable. * The risk model of the Society of Thoracic Surgeons (STS) uses an algorithm that is based on the presence of coexisting illnesses to predict 30-day operative mortality. The STS score equals the predicted mortality expressed as a percentage. A score of greater than 8% indicates high risk, 4%–8% intermediate risk, and less than 4% low risk (Dangas et al., 2020; Ishizu et al., 2021). ^†^ The CHA2DS2-VASc, is a measure of the risk of stroke among persons with atrial fibrillation. Weighted scores are based on the presence of congestive heart failure, hypertension, diabetes mellitus, or vascular disease; a history of stroke or transient ischemic attacks; an age of 65–74 years or 75 years or older; and sex. 1–2 points refer to low risk, 3–4 to moderate risk, and >5 to high risk (Jacobs et al., 2015; Van Mieghem et al., 2021). ^‡^ Combined endpoint event was defined as the composite of all-cause mortality, stroke, major bleeding, or any related clinical adverse events including acute kidney injury, coronary obstruction, major vascular complications, and valve dysfunction requiring reintervention.

Ten studies were published between 2017 and 2021, and one study was presented at the ACC Session in 2021. Among them, three were RCTs, three were prospective cohort studies, and the other five were retrospective cohort studies. Eight studies were conducted in Europe, one in Japan, one in the United States, and one from multiple countries. The range of the follow-up period was from 6 to 33.6 months.

Four studies included patients who were administered only a single kind of NOAC, and the remaining studies included patients administered various NOACs. The mean STS risk score ranged from 4.1 to 8.8, which indicates that most of the included patients were of moderate-to-high risk for cardiac surgery. The mean CHA_2_DS_2_-VASc score ranged from 4.6 to 5.6, also indicating that most patients were of moderate-to-high risk for stroke. Meanwhile, most of the included TAVR patients had various related diseases (Table 2).

**TABLE 2:** Related disease history of included patients.

Author	Group	N	Atrial fibrillation, %	Hypertension, %	Diabetes mellitus, %	Renal disease, %	Coronary artery disease, %	Stroke or intracerebral bleeding, %	Permanent pacemaker, %
Seeger et al. (2017)	NOAC	141	100	NA	32.6	44.7	66	11.3	16.3
	VKA	131	100	NA	32	48.9	58.8	14.5	13.7
Geis et al. (2018)	NOAC	154	94.2	95.5	30.5	NA	51.9	15.6	NA
	VKA	172	93.6	91.9	33.1	NA	51.2	14.5	NA
Jochheim et al. (2019)	NOAC	326	99.1	89.9	28.8	53.3	56.9	18.4	NA
	VKA	636	99.1	89.5	34.1	44.3	55.4	16.5	NA
Butt et al. (2021)	NOAC	219	100	87.2	17.8	5.9	54.3	34.8	NA
	VKA	516	100	88.6	24.2	14.2	54.5	25.2	NA
Kosmidou et al. (2019)	NOAC	155	100	91.7	35.3	8.9	76.3	22	NA
	VKA	778							
Kalogeras et al. (2020)	NOAC	115	68.7	NA	24.3	NA	13.8	NA	13
	VKA	102	59.8	NA	26.8	NA	15.2	NA	17.6
Kawashima et al. (2020)	NOAC	227	100	75.8	24.2	74.4	26	10.6	8.4
	VKA	176	100	76.7	24.4	77.8	35.2	19.3	10.2
Dangas et al. (2020)	NOAC	826	0	87.2	28.6	NA	39.3	6.2	9.7
	Antiplatelet	818	0	85.2	28.7	NA	37.3	4.3	9.8
Didier et al. (2021)	NOAC	1,378	70	NA	24.2	48.6	37.2	11.5	15.9
	VKA	1,093	70	NA	21.7	51.5	33.1	13.2	15.7
Van Mieghem et al. (2021)	NOAC	713	100	90.7	37.9	NA	41.1	17.3	NA
	VKA	713	100	92.1	36	NA	41.7	16.3	NA
Collet (2021)	NOAC	749	28.3	80.9	29.5	NA	52.3	10.4	NA
	VKA/Antiplatelet	228/523	26.5	80	28.5	NA	49.6	11.9	NA

NOAC: non-vitamin K oral anticoagulant; VKA: vitamin K antagonist; NA: not applicable.

In addition, the results of the risk of bias are demonstrated in Supplementary File S2. According to the Cochrane risk-of-bias tool for RCTs and the ROBINS-I tool for non-RCTs, only one observational study included in the meta-analysis was categorized as a moderate risk of bias; the others (consisting of three RCTs) were of a low risk of bias. For the three RCTs, randomized assignment with intention-to-treat analysis was used to lower the risk of bias in patient baseline characteristics such as age, sex, body mass index, valve type, risk score, and a history of related disease, as shown in Tables 1, 2. For the other six observational studies, the adjustment by propensity score matching was used in the analyses to prevent potential bias in the comparison of patient groups induced by confounders, as mentioned above (Kalogeras et al., 2020).

### Results of the Meta-analysis

**FIGURE 2:**
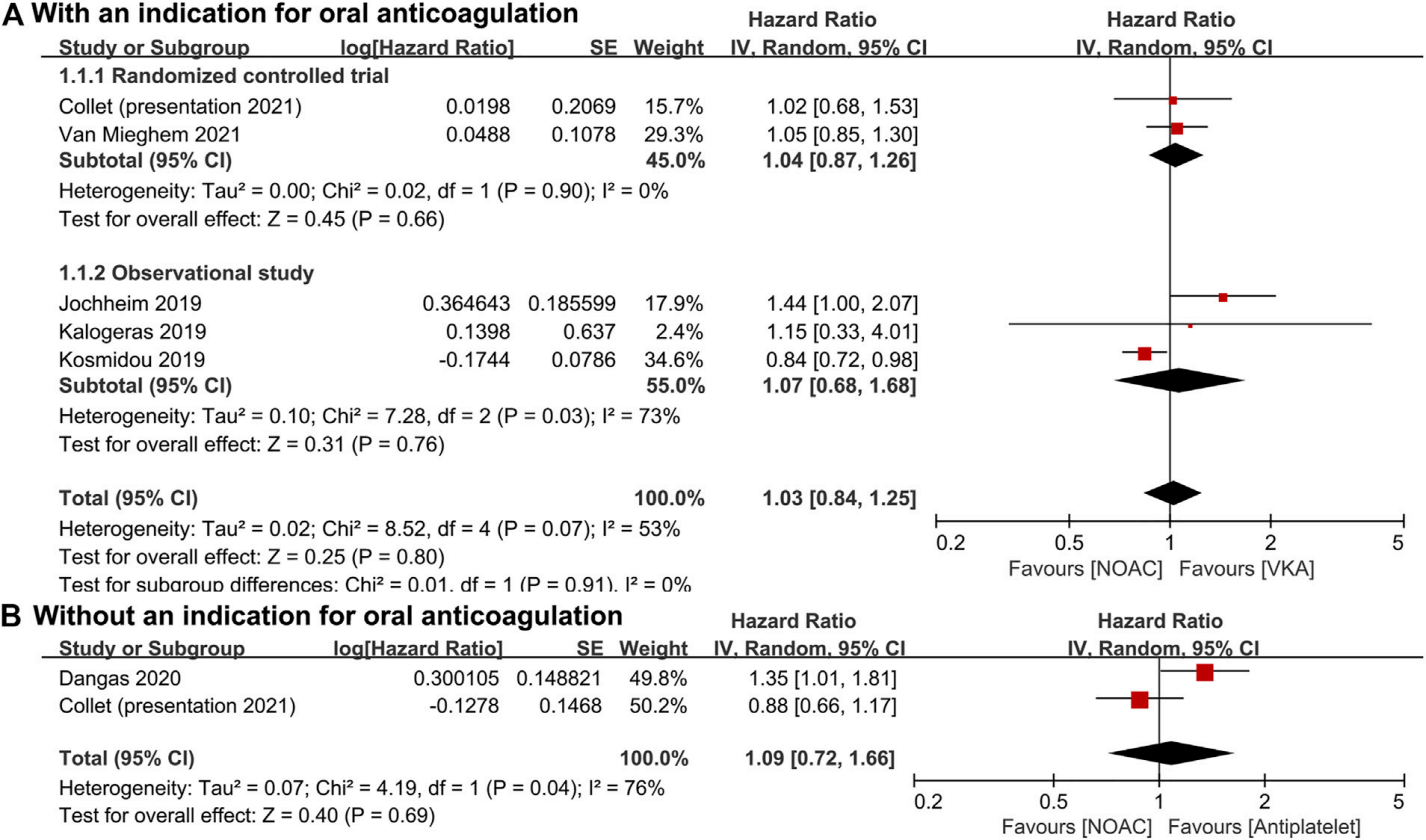
Forest plots for combined endpoint. **(A)**: a comparison of NOAC versus VKA in TAVR patients with an indication for oral anticoagulation; **(B)**: a comparison of NOAC versus antiplatelet in TAVR patients without an indication; NOAC: non-vitamin K oral anticoagulant; VKA: vitamin K antagonist; SE: standard error; CI: confidence interval.

**FIGURE 3:**
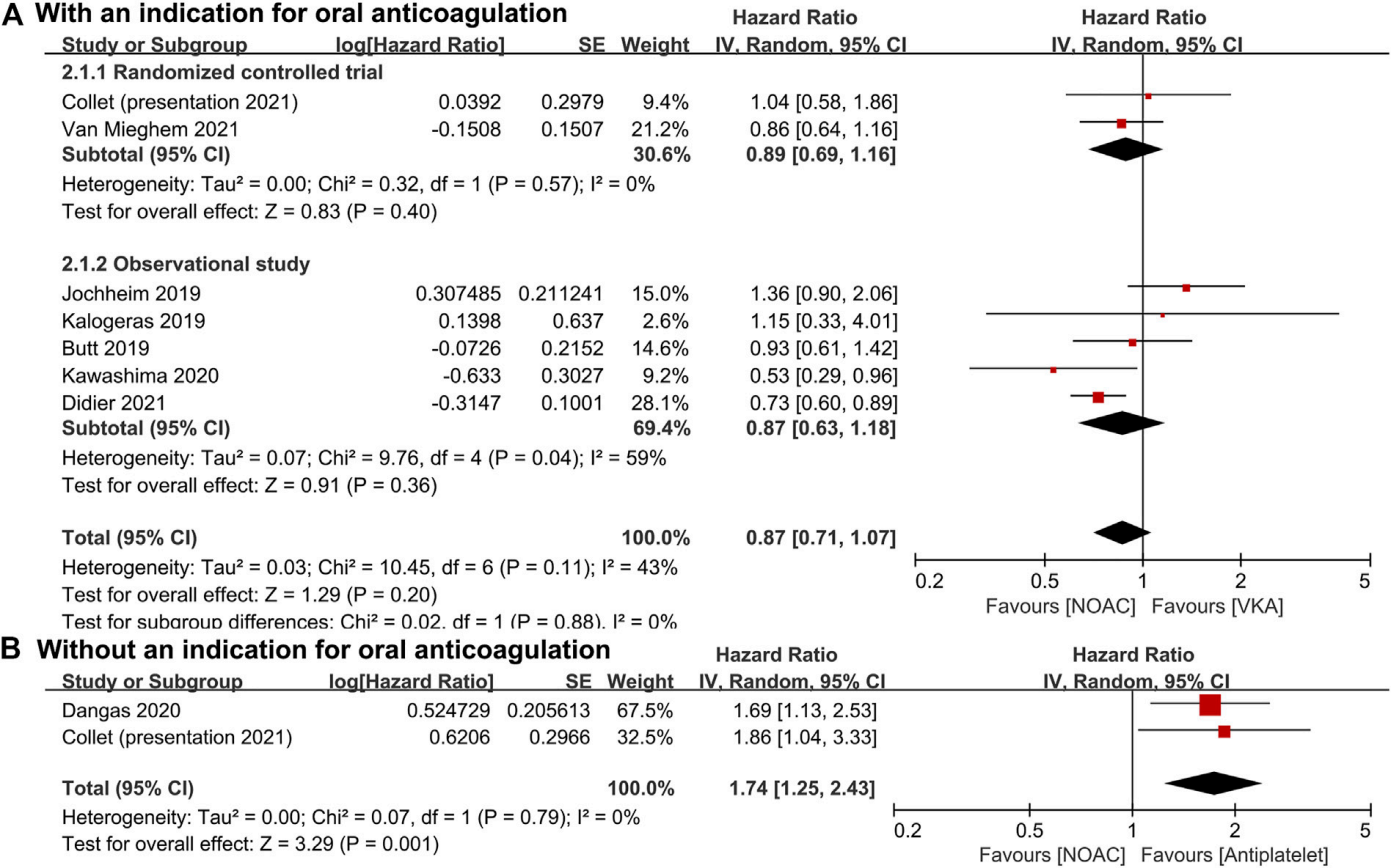
Forest plots for all-cause mortality. **(A)**: a comparison of NOAC versus VKA in TAVR patients with an indication for oral anticoagulation; **(B)**: a comparison of NOAC versus antiplatelet in TAVR patients without an indication; NOAC: non-vitamin K oral anticoagulant; VKA: vitamin K antagonist; SE: standard error; CI: confidence interval.

**FIGURE 4:**
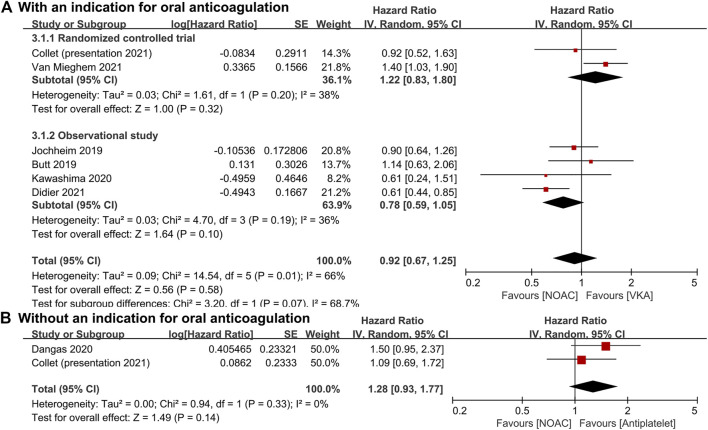
Forest plots for major bleeding. **(A)**: a comparison of NOAC versus VKA in TAVR patients with an indication for oral anticoagulation; **(B)**: a comparison of NOAC versus antiplatelet in TAVR patients without an indication; NOAC: non-vitamin K oral anticoagulant; VKA: vitamin K antagonist; SE: standard error; CI: confidence interval.

**FIGURE 5:**
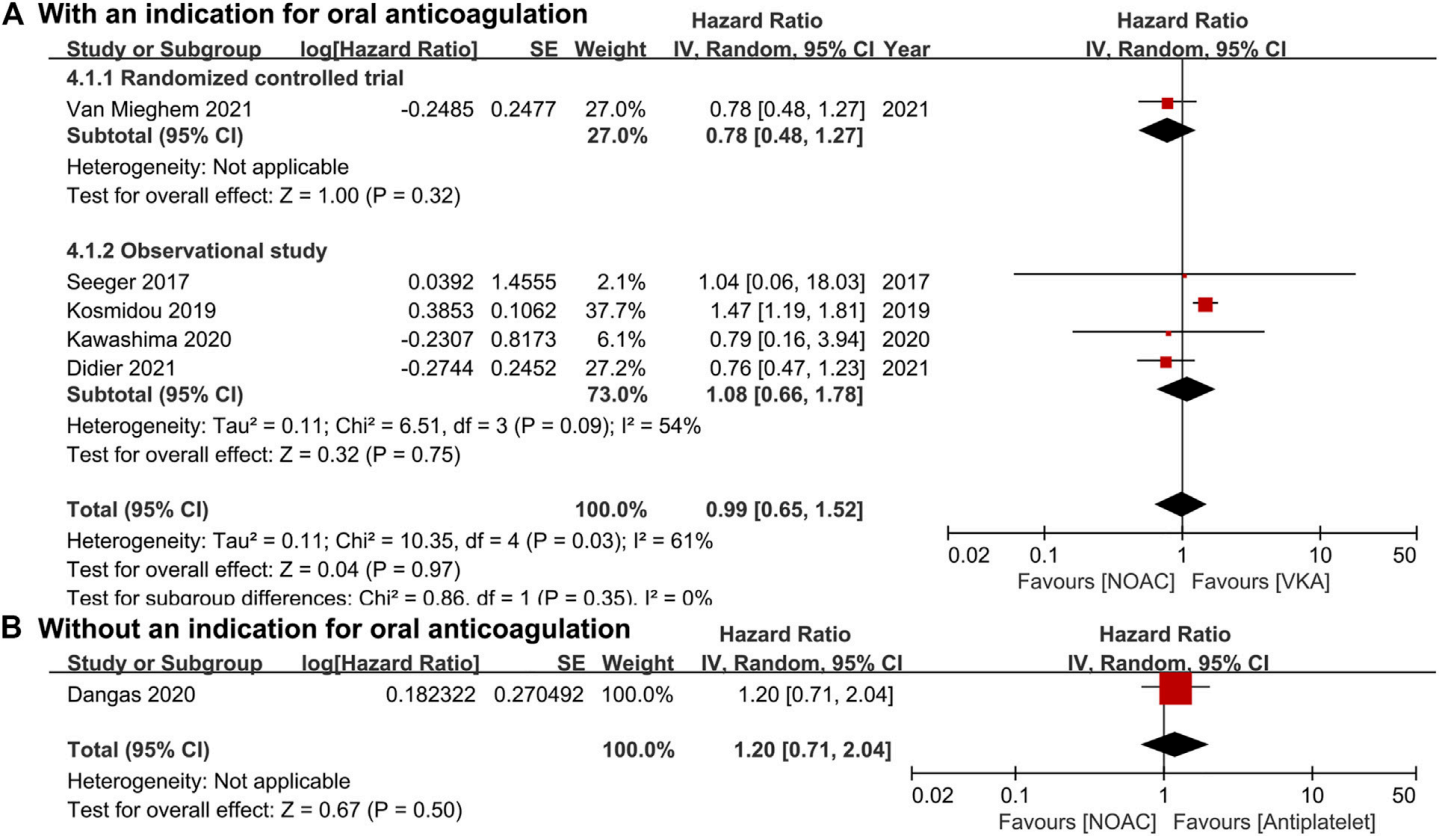
Forest plots for stroke. **(A)**: a comparison of NOAC versus VKA in TAVR patients with an indication for oral anticoagulation; **(B)**: a comparison of NOAC versus antiplatelet in TAVR patients without an indication; NOAC: non-vitamin K oral anticoagulant; VKA: vitamin K antagonist; SE: standard error; CI: confidence interval.

For patients with an indication for oral anticoagulation, there were no significant differences between the NOAC and the VKA groups in the total outcomes of the combined endpoint (HR 1.03, 95% CI 0.84–1.25, *p* = 0.80; Figure 2A), all-cause mortality (HR 0.87, 95% CI 0.71–1.07, *p* = 0.20; Figure 3A), major bleeding (HR 0.92, 95% CI 0.67–1.25, *p* = 0.58; Figure 4A), or stroke (HR 0.99, 95% CI 0.65–1.52, *p* = 0.97; Figure 5A). In addition, the results of the subgroup analyses by RCTs and observational studies were consistent with the above total outcomes.

For patients without an indication for oral anticoagulation, the pooled estimates of all-cause mortality (HR 1.74, 95% CI 1.25–2.43, *p* < 0.05; Figure 3B) favored antiplatelet rather than NOAC administration. In addition, the pooled estimates of the combined endpoint (HR 1.09, 95% CI 0.72–1.66, *p* = 0.69; Figure 2B), major bleeding (HR 1.28, 95% CI 0.93–1.77, *p* = 0.14; Figure 4B), and stroke (HR 1.20, 95% CI 0.71–2.04, *p* = 0.50; Figure 5B) showed no significant differences between the two groups. Because both included studies were RCTs, there was no subgroup analysis in the comparison.

### Sensitivity Analysis and Publication Bias

We conducted sensitivity analyses to ascertain the primary origin of heterogeneity. After temporarily omitting one study (Kosmidou et al., 2019; Kosmidou et al., 2019; Didier et al., 2021) from the combined analyses, we found that the pooled estimates of endpoint (HR 1.12, 95% CI 0.95–1.32, *p* = 0.19, I^2^ = 0%), major bleeding (HR 1.06, 95% CI 0.82–1.36, *p* = 0.67, I^2^ = 30%), and stroke (HR 0.77, 95% CI 0.56–1.08, *p* = 0.13, I^2^ = 0%) were still consistent with the former values (HR 1.03, 95% CI 0.84–1.25, *p* = 0.80, I^2^ = 53%, HR 0.92, 95% CI 0.67–1.25, *p* = 0.58, I^2^ = 66%, HR 0.99, 95% CI 0.65–1.52, *p* = 0.97, I^2^ = 61%, respectively). Therefore, we could consider these synthetic results stable and convincible. We planned to conduct a funnel plot with Egger’s regression test to detect the publication bias across the studies; however, none of the outcomes met the criteria of including a minimum of 10 studies.

## Discussion

This meta-analysis included RCTs and non-RCTs for the evaluation of comparisons of NOACs with VKA or antiplatelets in the long-term outcomes of patients undergoing TAVR with or without an indication for oral anticoagulation. We found no significant differences between the NOAC and the VKA groups in the combined endpoint, all-cause mortality, major bleeding, or stroke. However, we did observe significant differences in the all-cause mortality between the NOAC and antiplatelet groups.

We noticed that although Liang et al. (2020) and Ueyama et al. (2020) conducted two meta-analyses that compared NOACs with VKA in patients after TAVR, Liang et al. included seven studies of 5,089 patients for the meta-analysis, and they demonstrated a priority in VKA against NOACs in stroke (risk ratio 1.44, 95% CI 1.05–1.99, *p* = 0.02) (Liang et al., 2020). Ueyama et al. conducted a meta-analysis involving 2,569 patients from five studies and found that all-cause mortality (odds ratio [OR] 1.07, 95% CI 0.73–1.57, *p* = 0.72), major and/or life-threatening bleeding (OR 0.85, 95% CI 0.64–1.12, *p* = 0.24), and stroke (OR 1.52, 95% CI 0.93–2.48, *p* = 0.09) were similar between DOACs and VKA in patients undergoing TAVI with concomitant indication for oral anticoagulation.

Two points in our study were different from the above studies. First, to assess the follow-up outcomes related to time, we calculated the time-to-event data as the HR value. Second, we aimed to focus on the effects of NOACs on TAVR patients. Thus, we included studies involving NOAC administration, regardless of whether the patients did or did not have an indication for anticoagulation. As a result, two kinds of comparisons were performed based on whether patients had an indication for oral anticoagulation, respectively, that is, patients with an indication for oral anticoagulation (NOACs versus VKA) and patients without an indication (NOACs versus antiplatelet). Such two points make this study different from the previous ones, and we believe that the results of our study can supplement the conclusions of previous studies and provide evidence for clinical decision-making.

About the risk of bias, because of the utility of intention-to-treat analysis in RCTs and propensity score matching in most observational cohorts, the risks of bias were lowered, and nine of the included studies were of low risk of bias.

As the main outcome, the combined endpoint was mostly defined as the composite of all-cause mortality, stroke, major bleeding, and other critically relevant cerebrovascular events (Jochheim et al., 2019; Butt et al., 2021). The studies by Jochheim et al. (HR 1.44, 95% CI 1.00–2.07, NOAC vs. VKA), Kalogeras et al. (HR 1.15, 95% CI 0.33–4.04, NOAC vs. VKA), Van Mieghem et al. (HR 1.05, 95% CI 0.85–1.31, NOAC vs. VKA), and Collet et al. (HR 0.92, 95% CI 0.73–1.16, apixaban vs. standard of care) all showed that during the follow-up, there were no significant differences in the long-term endpoint between the NOAC and other groups (Jochheim et al., 2019; Kalogeras et al., 2020; Collet, 2021; Van Mieghem et al., 2021). However, Dangas et al. reported a higher risk of death or thromboembolic events (HR 1.35, 95% CI 1.01–1.81) in the rivaroxaban group as compared with the antiplatelet group (Dangas et al., 2020).

With regard to the risk of death, Butt et al. not only compared the all-cause mortality between NOACs and VKA (HR 0.93, 95% CI 0.61–1.40) but also performed subgroup analyses of dabigatran versus VKA, rivaroxaban versus VKA, and apixaban versus VKA and found no significant difference between any of them (Butt et al., 2021). Moreover, Jochheim et al. (HR 1.36, 95% CI 0.90–2.06) and Collet et al. (HR 1.04, 95% CI 0.58–1.86) after 1-year follow-up and Kalogeras et al. (HR 1.15, 95% CI 0.33–4.04) after 2-year follow-up also reported no significant differences in mortality between the NOAC and the VKA groups (Jochheim et al., 2019; Kalogeras et al., 2020; Collet, 2021). However, Kawashima et al. (HR 0.53, 95% CI 0.29–0.96) and Didier et al. (HR 0.73, 95% CI 0.60–0.89) demonstrated that as compared with VKA, NOACs might be associated with lower long-term mortality in TAVR patients with concomitant atrial fibrillation (Kawashima et al., 2020). In patients with nonvalvular atrial fibrillation, NOACs were also reported to be associated with a reduced risk of all-cause mortality as compared with warfarin, especially in Asian patients (Chan et al., 2018; Xue and Zhang, 2019).

Among patients who do not require oral anticoagulation, Dangas et al. reported a total of 64 deaths in the rivaroxaban group and 38 in the antiplatelet group, respectively (HR 1.69, 95% CI 1.13–2.53), indicating a higher mortality rate in the rivaroxaban group (Dangas et al., 2020). Meanwhile, Collet et al. also found a higher risk of all-cause death (HR 1.86, 95% CI 1.04–3.34), especially noncardiovascular death (HR 2.99, 95% CI 1.07–8.35), in the apixaban group compared with the antiplatelet group (Collet, 2021). Most deaths occurred long after the discontinuation of the trial drug and were due to noncardiovascular causes, such as sepsis or acute renal failure (Dangas et al., 2020; Collet, 2021). The mechanism of the higher mortality in the NOAC group remains unclear.

As one of the most important complications, major bleeding (including disabling and life threatening) was also use to assess the safety of NOACs and other antithrombotic agents. Major bleeding occurred in 46 patients in the NOAC group and 31 patients in the antiplatelet group, respectively (HR, 1.50; 95% CI, 0.95–2.37) according to Dangas et al. (Dangas et al., 2020). Although Butt et al. (HR 1.14, 95% CI 0.63–2.06), Jochheim et al. (HR 0.90, 95% CI 0.64–1.26), and Kawashima et al. (HR 0.61, 95% CI 0.25–1.52) also reported no increased risk of long-term major bleeding between the NOAC and the VKA treatments, Van Mieghem et al. (HR 1.40, 95% CI 1.03–1.91) showed higher risk and Didier et al. (HR 0.61, 95% CI 0.44–0.85) showed lower risk in the NOAC group (Jochheim et al., 2019; Kawashima et al., 2020; Butt et al., 2021; Didier et al., 2021; Van Mieghem et al., 2021). In patients with nonvalvular atrial fibrillation, NOACs were suggested with a decreased risk of major bleeding compared with VKA (Caldeira et al., 2015; Chan et al., 2018; Xue and Zhang, 2019). In patients with heart failure, apixaban might be associated with a comparable risk of major bleeding compared with aspirin, while other NOACs might be associated with a higher risk (Huang et al., 2020).

As another important complication after TAVR, stroke might occur due to the thrombosis in patients with low-intensity anticoagulation or no anticoagulation (Hansson et al., 2016; Otto et al., 2021). During the follow-up, incidences of both ischemic (HR 1.28, 95% CI 0.73–2.23) and hemorrhagic (HR 0.67, 95% CI 0.11–3.67) stroke did not differ significantly between the NOAC and antiplatelet groups in patients without an indication (Dangas et al., 2020). Similarly, in patients with an indication, the risks of all strokes were not significantly different between the NOAC and the VKA groups (Seeger et al., 2017; Kawashima et al., 2020; Didier et al., 2021; Van Mieghem et al., 2021). However, NOACs have been shown to be more effective than VKA for reducing the risk of stroke in patients with nonvalvular atrial fibrillation (Granger et al., 2011; Ajam et al., 2020; Diener et al., 2020).

In addition, although rivaroxaban and apixaban are considered to be associated with a lower risk of subclinical valve thrombosis (Dangas et al., 2020; Collet, 2021), because of the unexplained higher mortality, we, for now, cannot suggest NOACs as a routine antithrombotic therapy in patients who have undergone TAVR who do not require oral anticoagulation.

## Limitations

This study has several limitations. First, because of the limited number of included studies, there might be publication bias in our pooled estimates, and thus the results should be interpreted with caution. Second, the patients included in most studies were administered both anticoagulation and antiplatelet agents in the early term; therefore, the early-term outcomes might be affected by the unknown potential interaction. For this reason, we did not use the early-term but rather the long-term outcomes to assess the safety and efficacy of NOACs in TAVR patients. Third, a different category of NOACs and a different type of implanted bioprosthetic valve might be the origin of the heterogeneity; thus, to make the results more reliable, we performed analyses using a random-effects model. Fourth, although most included TAVR patients were of moderate-to-high risks for cardiac surgery and stroke, original studies did not separate them into two different risk subgroups, respectively. Therefore, this meta-analysis could not specifically check those high-risk patients.

## Conclusion

For patients with an indication for oral anticoagulation after TAVR, NOACs may be an alternative with noninferior outcomes to VKA. However, for patients with no indication, the use of an antiplatelet appears to be a safer choice, with a lower rate of all-cause mortality as compared with NOACs. Given that some limitations cannot be overcome, more high-quality studies and follow-up data are needed to confirm our findings.

## Data Availability

The original contributions presented in the study are included in the article/Supplementary Material, further inquiries can be directed to the corresponding author.
