# Supplementary material for: Non-Vitamin K Oral Anticoagulant After Transcatheter Aortic Valve Replacement: A Systematic Review and Meta-Analysis
Source: Front Pharmacol. 2022 Feb 11;13:755009. doi: 10.3389/fphar.2022.755009 (PMC8880334; doi:10.3389/fphar.2022.755009)
Supplement: Supplementary file 2 [file Table2.DOCX]

Supplementary File 2: Risk of bias of included studies

Content 1. Risk of bias of included randomized studies

| Author | Sequence generation | Allocation concealment | Blinding of participants and personnel | Blinding of outcome assessment | Incomplete outcome data | Selective outcome reporting | Other bias | Total bias |
| --- | --- | --- | --- | --- | --- | --- | --- | --- |
| Dangas | Low | Low | Low | Low | Low | Low | Low | Low |
| Van Mieghem | Low | Low | Low | Low | Low | Low | Low | Low |
| Collet | Low | Low | Low | Low | Low | Low | Low | Low |

**Content 2. Risk of bias of included non-randomized studies**

| Author | Bias due to confounding | Bias in participants selection | Bias due to departures from intended interventions | Bias in measurements of interventions | Bias due to missing data | Bias in selection of the reported result | Bias in measurement of outcomes | Total bias |
| --- | --- | --- | --- | --- | --- | --- | --- | --- |
| Seeger | Moderate | Moderate | Moderate | Unclear | Unclear | Low | Moderate | Moderate |
| Geis | Moderate | Moderate | Moderate | Unclear | Unclear | Low | Moderate | Moderate |
| Jochheim | Low | Low | High | Low | Low | Low | Low | Low |
| Butt | Low | Low | High | Low | Low | Low | Low | Low |
| Kosmidou | Low | Low | Moderate | Low | Low | Low | Low | Low |
| Kalogeras | Low | Low | High | Low | Low | Low | Low | Low |
| Kawashima | Low | Low | High | Low | Low | Low | Low | Low |
| Didier | Low | Low | High | Low | Low | Low | Low | Low |
